# Effects of digital transformation and environmental resource integration capability on medical equipment suppliers’ green innovation performance

**DOI:** 10.1038/s41598-023-44274-5

**Published:** 2023-10-16

**Authors:** Qiong Wu, Shihan Wang, Anning Zhou, Bing Xia, Lucille Aba Abruquah, Zhen Chen

**Affiliations:** 1 School of Management Science and Engineering, Chongqing Technology and Business University, Chongqing, China; 2 Chongqing Research Center for Industrialization and Informatization Integration and Logistics Information Technology Application, Chongqing, China; 3 Postdoctoral Research station, Changzufeiyue Technology Development Co., Ltd, Chongqing, China; 4 School of Business Administration, Chongqing Technology and Business University, Chongqing, China; 5 CERATH Development Organization, Accra, Ghana; 6 Research Global, Accra, Ghana; 7 Dazhou Central Hospital, Dazhou, China

**Keywords:** Behavioural ecology, Environmental economics, Environmental social sciences

## Abstract

In today’s digital age, the effort of medical enterprises towards green innovation has gained traction in academic and business circles. However, the current research system for medical equipment suppliers lacks a systematic study on how digital transformation can enhance the outcomes of green innovation. This research aims to develop a theoretical framework for digital transformation, environmental resource integration capability, managerial environmental concern and green innovation performance with respect to the resource-based view and conducting empirical analysis using survey data from medical equipment supply enterprises. Our findings reveal that digital transformation has a significant effect on the promotion of green innovation performance through environmental resource integration capability. Moreover, the managerial environmental concern moderates above mediation effect. These findings not only provide compelling insights into the impact of digital transformation on green innovation performance but also have important implications for sustainable development and cleaner production relevant academic research and policy-making.

## Research Background

The widespread adoption of digital technology across various industries has resulted in digital transformation becoming a subject of considerable interest and research in both academic and business spheres^1^.The Chinese government's strong advocacy for environmental protection and green innovation in recent years has been met with active responses from the news media, government agencies, and various sectors of society. The increased emphasis on environmental protection has created a demand for medical equipment suppliers to make necessary adjustments and enhance their green innovation performance (GIP) in response to societal demands. Digital transformation (DT) is a kind of organizational change which refers to the utilization of novel digital systems, including artificial intelligence, the internet of things (IoT), cloud-based solutions, block-chain as well as mobile devices to achieve significant advancements in business operations, facilitate operational efficiency, or innovate new business models to improve customer experience^2^. It requires a corporate mindset that affects all functions and departments within an organization^3^. For instance, medical equipment suppliers can leverage digital transformation to gather data on environmental protection, make informed decisions that align with sustainable development, increase investment in green innovation, and enhance their performance in this area. However, the current research system for medical equipment suppliers lacks a systematic study on how digital transformation, environmental resource integration capability, and the managerial concern for the environment can enhance the effectiveness of green innovation. Such research is likely to have significant implications for the sustainable development and green innovation performance of medical equipment supplies enterprises, providing valuable insights and guidance. With this in mind, this study probes into the relationship between DT and GIP, assessing the mediating function of environmental resource integration capability (ERIC) as a moderator of managerial environmental concern (MEC). The research findings will contribute to an advanced understanding of the role of DT on the GIP of medical device suppliers for academic, business and government sectors. The study will provide benefits for the sustainable development of medical equipment supply enterprises by offering insights into the acquisition of green innovation-related technologies, knowledge and other resources. This, in turn, will enhance green innovation performance and have significant theoretical and practical value.

The study’s primary contribution is threefold. Firstly, it examines the influence of DT on GIP using a substantial amount of data. According to the research by Mo et al.^4^, green innovation performance refers to the ability and actual performance of using new technologies or other methods in new products and new businesses to enhance environmental benefits. Prior research has shown that digital transformation can help medical equipment supply enterprises revamp their strategies, establish new business models, enhance their green innovation performance, which has become a hallmark of many enterprises^5^. Academics have extensively studied the correlation between digital transformation and enterprise revolution and transformation as a crucial means of changing business modes. As highlighted by Nambisan et al.^6^, the development of advanced digital systems and infrastructure has led to notable transformations in the area of innovation and entrepreneurship. Additionally, Hinings et al.^7^ observed that digital transformation, which involves an integration of multiple digital innovations, introduces new players, structures, practices and values to enterprises, which is conducive to changing and supplementing existing game rules in the organizational field. Additionally, Chan^8^ referred to digital transformation as a business operation model which fundamentally alters the way enterprises operate by leveraging innovative digital technology and delivery technology to support the overall business. The existing research is rich in content in the digital field, however, few studies have explored the digital transformation of medical equipment and its impact on green innovation. Given the importance of medical equipment supplies to medical care and social livelihood, it is salient to delve deeper into the effect of DT on GIP of medical equipment suppliers. Therefore, this study uses survey data from medical equipment supplies enterprises to explore the influence of DT on GIP, which partially addresses the limitations of existing studies.

Secondly, this paper identifies ERIC as a mediator between DT and GIP. Environmental resource integration capability is the ability to identify, select, configure and utilize resources related to green production or environmental protection. Existing studies by ERIC, such as Yang et al.^9^ reported that preemptive environmental strategies have a greater positive effect on the ability of stakeholder to integrate than their ability to innovate. García-Sánchez et al.^10^ investigated the influence of various factors, including the environment, stakeholder integration ability, absorbability and technical skills impact business entrepreneurship, as well as the effect of business entrepreneurship on organizational performance. Shen et al.^11^ adopted the research framework of opportunity—resource integration, and examined how adaptive marketing capability (AMC) is linked to the utilization of opportunities and sustainable innovation performance (SIP). However, few of these studies mentioned the role and impact of ERIC on DT and GIP. Through ERIC, DT can modify green innovation strategy, reduce the risk and cost of green innovation, and enhance the value of green innovation, thereby improving the green innovation performance of medical equipment supply enterprises. As green innovation strategy has been found to have a beneficial influence on exploitative and exploratory types of green innovation alike^12^, this paper examines the mediating role of ERIC, which serves as a valuable addition to the current body of research.

Finally, with reference to the Resource-based view (RBV), this study found that managerial environmental concern plays positively moderates roles in the models of DT-ERIC and ERIC-GIP. According to Song et al.^13^ and Mo et al.^4^, MEC refers to the degree of concern for environmental protection in firm's management or business activities. Early researches have also revealed that MEC is linked to GIP. For instance, Saudi et al.^14^ used two forms of green innovation and multiple indicators to determine the MEC. Yusoff et al.^15^ also investigated the link between environmental performance and practices including recruiting and selecting environmentally-conscious employees, providing training and development programs focused on environmental awareness and sustainability, and offering compensation incentives for environmentally-friendly practices based on resource-based views and theories. Additionally, Seman et al.^16^ pointed out that organizations are increasingly adopting environmental management initiatives including growing green supply chain management (GSCM) and environmentally-friendly innovation, as a result of the promotion of environmental protection awareness among the public and the enforcement of government regulations. From the perspective of RBV, managerial environmental concern can help to draw the attention of medical equipment suppliers towards environmental innovation, identify management issues, and increase support of environmental resource integration in management and strategic decision-making. Additionally, this research examines the moderating effect of MEC in DT-ERIC and ERIC-GIP. Moreover, examines the moderated mediation effects of DT-ERIC-GIP. This study expanded the use of the RBV in the area of medical equipment supply.

In conclusion, based on the RBV, this study establishes a theoretical model known as "DT-ERIC-GIP", elucidates the mediating mechanism, and explores the moderating effect of MEC on the model. This study therefore holds significant theoretical and practical importance.

## Framework and hypotheses

### Framework Development

In the era of digital transformation, medical equipment providers must adapt to keep up with the innovation. The convergence of traditional medical devices with high-tech consumer electronics is leading to the development of smarter, customizable and connected products. While conventional companies leverage the ability to generate value within their organization or supply chain, digital systems rely on a collaborative ecosystem facilitated by a platform to engage with a broader range of partners and participants and create value through coordinated efforts^17^. The integration of digital transformation in the medical equipment supply sector is occurring silently amidst the ongoing era of innovation, as its adoption is rapidly gaining momentum. The adoption of digital transformation in medical equipment supply has stimulated managerial environmental concern. Due to the demand and appeal from various sectors of society, the academic community has also started to discuss the issues related to the digital transformation of medical equipment supply. For instance, Ângelo et al.^18^ pointed out that the new service is backed by dynamic QR code recognition and mobile health technology. The digital ecosystem presents opportunities for improving the quality of drug information and reconnecting drug customers with drug manufacturers. Jahankhani and Kendzierskyj^19^ also pointed out that in order to achieve several benefits, digital transformation must go beyond mere implementation and ensure secure and protected data exchange where several benefits can be obtained. With the growing risk of data breaches, the healthcare industry is exploring the potential benefits of block-chain technology in greater depth. Block-chain technology offers a mechanism to ensure invariance, audit trails, security, and data privacy protection, thus contributing to the improvement of healthcare transformation. Smith et al.^20^ highlighted that the healthcare supply chain (HSC) ensures the safety and quality of healthcare services by providing healthcare organizations with Clinical Safety Evaluation Model (CSEM) through the digital transformation of its supply chain. CSEM applies a modified version of the information success models originally proposed by DeLone and McLean, along with established safety standards within the healthcare supply chain domain. While these scholars have studied issues related to digital transformation in medical equipment supply, there remains a research gap on the impact of DT on the GIP of medical equipment supply enterprises, especially regarding the influencing mechanism. Thus, there is a need to conduct a comprehensive investigation and analysis. To address these issues, this study argues that the RBV perspective provides a theoretical framework of digital transformation, environmental resource integration capability, managerial environmental concern and green innovation performance, and have empirically tested it using investigation data of a large medical equipment supply enterprise.

Specifically, based on the RBV, the focus of this research is to investigate the correlation between DT, ERIC, MEC and GIP. The research first examines the role and effect of ERIC in the correlation between DT and GIP. The findings depict that the combination of green capability and green business strategy has a positive effect on the creation of green and the development of environmentally-friendly innovation. Moreover, the creation of green value through collaboration enhances the green performance of enterprises and facilitates enterprises’ green innovation. The incorporation of ERIC not only enables enterprises to achieve green technology performance but also improves the creation of green value. Furthermore, the creation of green value through collaboration serves as a moderator in the correlation between comprehensive capability and environmentally-friendly innovation^21^. Using causal logic, it can be concluded that the environmental resource integration capability of medical equipment supply enterprises is a key factor in their green innovation performance. Also, managerial environmental concern plays an important role in determining corporate behavior performance. This study thus concludes that the effect of DT on ERIC and GIP can be influenced by managerial environmental concerns.

**Figure 1:**
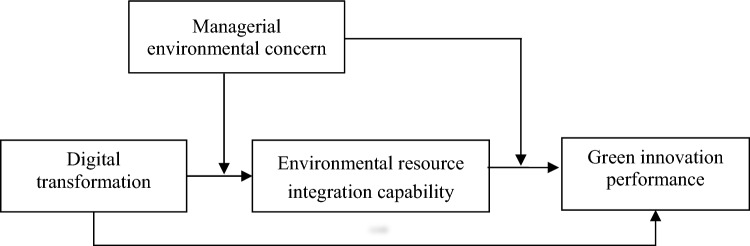
Research framework.

In summary, Fig. 1 depicts the research framework presented in this paper.

### The relationship between digital transformation and green innovation performance

There is currently no unified definition for the concept of “green innovation performance”. Scholars such as, Du et al.^22^, Albort-Morant et al.^23^, and Yang and Lin^24^ have used the term in their research. For instance, according to Du et al.^22^, there is a positive correlation between green customer, supplier integration and the performance of environmentally-friendly innovation. In the manufacturing industry, Albort-Morant et al.^23^ recommended that managers prioritize the strengthening of absorptive capacity to foster the generation of novel information and promote GIP. Meanwhile, Yang and Lin^24^ emphasized the importance of collaboration within the supply chain to attain green innovation performance. This paper defines GIP as the implementation of eco-friendly design, innovative processes, and management practices by organizations, to address environmental challenges and attain specific environmental protection and sustainable development goals. It is an evaluation index of the economic and social value added to an enterprise through the development or adoption of new technologies.

From the Resource-based view (RBV) perspective, medical equipment supplies companies undergoing GIP by utilizing various of digital technologies, including big data, artificial intelligence, cloud-based solutions, block-chain and IoT. The effect of DT on environmental protection can be seen primarily in two ways. On the one hand, DT facilitates the collection of environmental protection-related information. This enables enterprises gain a better understanding of the demands of news media, government, society and customers. Based on this understanding, enterprises can make strategic decisions that align with sustainable development principles. This can also motivate them to enhance their investment in green technology to enhance GIP. On the other hand, the use of DT can foster enterprises to easily access relevant external information to better understand the needs of stakeholders and make sustainable decisions. This is instrumental in addressing the problem of information asymmetry in environmental protection technology by facilitating the acquisition of green innovation-related technologies, knowledge and other resources. This also enables medical equipment supplies enterprises to utilize new technology to undertake scientific environmental demands analysis^25^, effectively change the inefficient supply of medical equipment model^26^, to optimize medical resources utilization^27^, eventually to promote medical equipment supply enterprises’ performance of green innovation^28^.

Based on this, we propose the following hypothesis:

#### H1

Digital transformation has a positive effect on green innovation performance.

### The mediating effect of environmental resource integration capability

With the rising importance of big data and digital technology, there has been growing emphasis on GIP and changes in the business models of medical equipment companies, which has enabled them to quickly adapt to changing market demands.

From the resource-based view (RBV) perspective, the adoption of digital transformation in medical equipment supply enterprises can benefit them in several ways. This includes improving their ability to analyze large datasets, gaining real-time insights, and promoting research and innovation, leading to positive impacts as suggested by^29^. These benefits can be observed in two main aspects. Firstly, the Digital transformation promotes the utilization of digital technologies resource in medical equipment supply companies^30^. Additionally, the focus of government and customs authorities on environmental protection has led to the application of cutting-edge technologies like big data, cloud computing, and artificial intelligence, allowing enterprises to search for and efficiently identify the relevant environmental resources^31^. Secondly, the adoption of digital transformation helps improve advanced innovations like big data, cloud computing and artificial intelligence. This is conducive to improving environmental resource integration capability^32^.

Based on this, we propose the following hypothesis:

#### H2

Digital transformation has a positive effect on environmental resource integration capability.

The health care service system relies significantly on medical equipment, which is essential to the global market^33^. Kuo et al.^34^ utilized the RBV and resource dependence theory (RDT) to explore the correlation between favorable environmental strategy, environmentally sustainable innovation, eco-friendly competitive edge and green core competitiveness in the hotel industry. Their findings revealed that positive environmental strategy has a favorable impact on ecological innovation, which subsequently impacts the green core competitiveness. The study also revealed that environmental resource integration capacity, which encompasses crucial elements of environmental innovation, extends to both their green innovation performance and medical equipment supply enterprises, enhancing their green innovation performance. Specifically, environmental resource integration capability promotes the green innovation performance of medical equipment supply enterprises in two ways. Firstly, this capability enables enterprises to identify key resource of green innovation such talent, technology, demand information, and policies supporting environmental protection^35^. As a result, medical equipment suppliers can leverage this resource to enhance their green innovation performance. Secondly, environmental resource integration capability can also facilitate the efficient utilization of existing environmental technologies, funds, talents and other resources, thereby improving the efficiency of green innovation, and ultimately enhancing the GIP of companies. For instance, Miao et al.^36^ revealed that advancement in green technology results in a relatively high level of efficiency in utilizing natural resources, with an increasing trend. The efficient utilization of natural resources creates a conducive environment for the selection of the best innovation behavior, enabling rational use of natural resources to support economic development and environmental protection. Based on these observations, we suggest the following hypothesis:

#### H3

Environmental resource integration capability is positively correlated to green innovation performance.

Reis et al.^37^ find that digital revolution has become beneficial across all aspects of business operations, necessitating managers to modify their strategies in light of the new digital landscape. Environmental resource integration capability may be a crucial factor for organizations to adjust to this change. Sun and Guo^38^ pointed out that green innovation can be considerably boosted through digital transformation by strengthening enterprises' green innovation capability and reducing internal and external costs. This, in turn, encourages enterprises to facilitate resource exchange among stakeholders through environmental resource integration capability. This approach minimizes the risks and expenses involved in green innovation, increasing the value of green innovation, and ultimately promoting improvement in the GIP of medical equipment suppliers. Additionally, the integration of environmental resources during the digital transformation process enables managers to obtain key internal and external information, helping medical enterprises in adjusting green innovation approach and strengthening their green innovation ability to achieve GIP.

In line with these findings, we hypothesize that:

#### H4

Environmental resource integration capability mediates the relationship between digital transformation and green innovation performance.

### The moderating role of MEC on DT and ERIC

The effect of DT on ERIC has been a widely researched topic among academics and the business community. For instance, Wu et al.^39^ elucidated that digital capabilities can enable open innovation, but this phenomenon is influenced by various determinants both within and outside the organization, and the interaction between environmental variations and managerial digital consciousness plays a catalytic role. Additionally, Bocconcelli et al.^40^ emphasized that technology has become a key operational resource in the transformation of resource integration mode. Furthermore, Mengcheng and Tuure^41^ argued that the use of information technology in collaborative development processes fosters social contact and enables the integration of resource. Under the background of digital transformation, managerial environmental concern plays a vital role in providing channel knowledge (one kind of resource) spillover effects, and integrating environmental resources. Thus, low managerial environmental concern in medical equipment supply enterprises weakens the efficiency of relevant knowledge resource absorption, transformation and transmission, leading to reduce the effects of digital transformation on environmental resource integration capability. Conversely, high managerial environmental concerns enhance the efficiency of relevant knowledge resource absorption, transformation and transmission, resulting in increased knowledge resource spillover effect due to digital transformation. This in turn, promotes the enhancement of environmental resource integration capability of medical equipment supply enterprises.

In line with these findings, we hypothesize that:

#### H5

Managerial environmental concern positively moderates the relationship between digital transformation and environmental resource integration capability.

### The moderating effect of MEC on ERIC and GIP

With the rapid advancement of digital technology and its widespread adoption across industries has brought attention to the significance of the integration of environmental resources in enhancing green innovation performance. Consequently, this has become a trending research topic and area of interest across various sectors. For instance, Wu et al.^39^ highlighted that enterprises are proactively seeking out and acquiring digital resources, leveraging their digital capabilities to manage and utilize these resources effectively, and using digital technology to foster open innovation. Qiu et al.^42^ also emphasized the relevance of the integration of environmental resources, reconstruction of resource and environmental awareness skills within the framework of environmental agility, as a mediator between green product development and competitive advantage. Furthermore, Dangelico et al.^43^ pointed out that a company’s ability to adapt and respond to sustainability challenges involves three core processes: integrating external and internal resources and constructing and reallocating resources. These processes considerably impact transforming and improving the company’s regular sustainability capabilities and its ability to innovate with environmentally friendly solutions. Managerial environmental concern has drawn the attention of medical equipment suppliers towards environmental innovation and bolstered their support for the integration of environmental resources in the aspects of management and enterprise strategic decision-making. This enhances the channel knowledge for environmental resource integration capability, thereby amplifying its impact on green innovation performance for medical equipment suppliers. Therefore, when managerial environmental concerns are low in medical equipment supply enterprises. As a result, the amplification effect on environmental resource integration capability is small, which results in minimal impact on green innovation performance. Conversely, higher managerial environmental concerns of medical equipment supply enterprises expand the scope of knowledge acquisition and increase heterogeneity. This in turn increases the impact on environmental resource integration capability, resulting in an increased influence green innovation performance.

With respect to this, we propose the following hypothesis:

#### H6

Managerial environmental concern positively moderates the relationship between environmental resource integration capability and green innovation performance.

## Method

### Variables

To analyze the variables of interest, a 7-point Likert scale was utilized in this study.

#### The dependent variable

Green innovation performance, was measured based on four aspects, namely raw materials, energy-saving, waste disposal, and recyclability. Drawing on insights from prior research by Song et al.^13^, Mo et al.^4^ and enterprise interview data, this study included the following aspects to assess GIP: (1) Our company has introduced new products or services that employ environmentally friendly materials; (2) Our company has introduced to new products or services reduce energy consumption; (3) Our company has developed a new product or business that promote pests control or engage proper disposal of waste materials; (4) Our company has developed new products or businesses that involve the recycling and reuse of all sold goods.

#### The independent variable

Digital transformation was measured based on the definition proposed by Warner and Wäger^2^ and Yao et al.^44^ and the classification criteria of Vial^45^. The measurement of digital transformation involved four aspects, including: (1)Significant changes in our company’s organizational structure and culture significantly driven by digital technology over the past three years; (2) Significant changes in our company's internal leadership style and staff skills driven by digital technology over the past three years; (3) Significant changes in our company’s operating process and sales channels driven by digital technology over the past three years; (4) Significant improvements in our company’s ability to adapt to environment driven by digital technology over the past three years.

#### The mediating variable

According to the definition of resource integration, we define environmental resource integration capability as the ability to identify, select, configure and utilize resources related to green production or environmental protection. The following indicators were used to measure environmental resource integration capability: (1) Our company possesses the capability to effectively identify resources such as, theoretical knowledge, application technology, management concept and partners that are conducive to environmental improvement; (2) Our company gives priority to resources such as, theoretical knowledge, applied technology, management ideas and partners that are conducive to environmental improvement; (3) Our company has rationally allocated resources, such as theoretical knowledge, application technology, management concept and partners, which are conducive to environmental improvement; (4) Our company makes reasonable use of the theoretical knowledge, applied technology, management ideas and partners that are conducive to environmental improvement.

#### The moderating variable

Managerial environmental concern was measured using the study by Song et al.^13^ and Mo et al.^4^. Thus, four measurement items were designed to assess managerial environmental concern: (1) Our company places great importance to innovation in environmental protection technology or concepts; (2) Our company is willing to actively provide resources needed for environmental protection; (3) Our company believes that pro-environment strategies will positively impact business development; (4) Our company attaches great importance to and is more willing to conduct business cooperation with pro-environment suppliers.

#### The control variables

Refer to the researches by Tang et al.^46^ and Xie et al.^47^, this study control the variables of firm age (Age), firm size (Size), Net profit (Np), R&D intensity (R&D), government subsidies (GS) and ownership. The firm age, net profit and government subsidies were directly measured. The size of the company was determined based on the number of employees. To assess the ownership structure, a dummy variable was used, with 1 indicating a private enterprise and 0 indicating state-owned enterprise. The level of R&D investment intensity was quantified as the ratio of R&D investment to sales.

### Data collection

This study obtained approval from the Academic Ethics Committee of DZ central hospital (a well-known large tertiary A hospital in China, Annual revenue of approximately $250 million) and fully complied with the guidelines of the ethics review board. This study utilized a questionnaire survey to gather data from medical equipment suppliers. Before filling out the questionnaire, the informed consent was obtained from all subjects and we informed all respondents of the purpose of this study.

Following the design and testing of the questionnaire, we distributed it to suppliers of two third-class hospitals in western China. The preliminary data analysis yielded satisfactory results. Based on this, from January to March 2023, we collected data from senior executives of medical equipment supply companies using alumni platforms for MBA, EMBA and DBA. In total of 545 questionnaires were distributed, 346 questionnaires were collected, and 338 sample data were used for the data analysis, resulting in an effective response rate of 62.02%.

### Data analysis

We used regression analysis to explore the correlation between the study variables, and primarily utilized SPSS, PROCESS and AMOS for data analysis.

#### Reliability and validity of the questionnaire

In accordance with the research of Tang et al. (2023), this study carried out a unidimensional test and a common method deviation test. The principal component analysis results using the maximum variance method revealed that the first four factors explained 79.72% of the total variation. Additionally, the components matrix results showed a one-to-one correspondence between latent variables and measured variables depicted in Table 1. These findings indicate that the questionnaire used in this study has a single dimension. Additionally, results from Harman's single factor analysis depicted that the initial common factor accounted for 29.91% of the total variance. The percentage falls short of the 30% threshold set by some similar studies (while others require less than 50%). This suggests that there was no significant common methodological bias in this study.

**Table 1:** Components matrix.

Variables	Items	Components matrix			
		1	2	3	4
DT	Q1	.104	.865	.194	.028
	Q2	.163	.847	.185	.069
	Q3	.163	.830	.217	.053
	Q4	.131	.899	.142	.049
MEC	Q5	.088	.016	.021	.845
	Q6	.094	.028	.035	.871
	Q7	.075	.081	.006	.863
	Q8	-.038	.050	.016	.872
ERIC	Q9	.871	.167	.181	.064
	Q10	.874	.106	.246	.058
	Q11	.885	.174	.207	.039
	Q12	.848	.135	.220	.094
GIP	Q13	.229	.178	.869	.047
	Q14	.248	.179	.834	.029
	Q15	.171	.200	.880	.012
	Q16	.224	.222	.813	.000

This study also conducted factor analysis and reliability analysis. Table 2 presents results indicating that Cronbach's alpha and KMO values of the selected variables exceeded the recommended threshold 0.7. Additionally, AVE values were greater than suggested limit of 0.5, and CR values higher than the suggested limit of 0.7. These findings demonstrate that the study questionnaire has reliability and good convergent validity.

**Table 2:** Factor analysis and reliability analysis.

Variables	Cronbach’s alpha	KMO	AVE	CR
DT	0.915	0.854	0.7963	0.9399
MEC	0.888	0.841	0.7496	0.9229
ERIC	0.931	0.861	0.8273	0.9504
GIP	0.920	0.849	0.8072	0.9436

Furthermore, confirmatory factor analysis was performed to evaluate.

the validity of the study questionnaire. The findings obtained from the analysis in AMOS showed that the CMIN/DF value was 0.985, which is below the suggested critical value of 3. Also, the RMSEA value was close to 0, which is much less than the suggested threshold of 0.05. The standardized RMR value was 0.0278, which is less than the critical value of 0.05. Additionally, the CFI, TLI, IFI were all close to 1, while GFI and NFI were 0.966 and 0.976 respectively, all greater than 0.9. These findings demonstrate that the questionnaire employed this research has sound structural validity.

#### Descriptive statistics and correlation analysis

Table 3 shows the mean, standard deviation, highest and lowest values, and correlation analysis of the study variables. The findings revealed a statistically significant correlation between DT and GIP with a coefficient of 0.443 (*p* < 0.01). Additionally, the correlation analysis showed that the coefficient between DT and ERIC was 0.357 (*p* < 0.01) and the coefficient for ERIC and GIP was 0.488 (*p* < 0.01). These findings indicate that the sample data used in this study conform to the model setting.

**Table 3:** Descriptive statistics and correlation analysis.

Variables	1	2	3	4	5	6	7	8	9	10
1. Age	1									
2. Size	.081	1								
3. Np	.091	.141**	1							
4. R&D	.044	.090	.200**	1						
5. GS	−.001	.130*	.138*	.215**	1					
6. Ownership	−.112*	−.083	−.128*	−.085	−.077	1				
7. DT	−.004	.187**	.116*	.099	.184**	−.136*	1			
8. MEC	.048	.038	.081	.121*	.044	−.030	.119*	1		
9. ERIC	−.004	.171**	.220**	.226**	.237**	−.166**	.357**	.144**	1	
10. GIP	.051	.292**	.266**	.229**	.287**	−.227**	.443**	.072	.488**	1
Min	1.000	1.000	1.000	1.000	1.000	0.000	1.000	1.000	1.000	1.000
Max	5.000	5.000	5.000	5.000	5.000	1.000	7.000	7.000	7.000	7.000
Mean	2.926	2.524	2.790	2.858	2.698	0.680	4.127	4.295	4.036	4.237
SD	1.424	1.339	1.374	1.449	1.453	0.467	1.740	1.650	1.866	1.735

The Minimum and Max are the mean values of the combined calculation of the latent variables corresponding to the variables. *p < 0.05; **p < 0.01; Two-tailed.

## Result

### Regression analysis of main effect and intermediate effect

The findings from the regression analysis for both the direct effect and mediating effect models in this study are shown in Table 4. Models M1 to M4 have GIP as the dependent variable, whereas models M5 to M6 have ERIC as the dependent variable. The highest VIF in each model is all less than 10, indicating that there was no significant issue of multi-collinearity. Additionally, the R2 and F values, along with other essential parameters, indicate that the model has a strong level degree of fit.

**Table 4:** Regression analysis of principal effect and intermediate effect.

Variables	Dependent variable: GIP				Dependent variable: ERIC	
	M1	M2	M3	M4	M5	M6
Age	− 0.005 (0.06)	0.007 (0.055)	0.012 (0.055)	0.017 (0.053)	− 0.045 (0.068)	− 0.035 (0.065)
Size	0.22 (0.064) ***	0.168 (0.06) ***	0.179 (0.059) ***	0.149 (0.057) ***	0.111 (0.072) *	0.068 (0.07)
Np	0.164 (0.063) **	0.144 (0.059) **	0.112 (0.059) *	0.108 (0.057) *	0.142 (0.072) **	0.126 (0.069) *
RD	0.121 (0.06) *	0.109 (0.056) *	0.068 (0.056)	0.070 (0.054)	0.145 (0.068) **	0.135 (0.066) **
FS	0.197 (0.06) ***	0.149 (0.056) **	0.137 (0.056) **	0.114 (0.054) *	0.163 (0.068) **	0.123 (0.066) *
Ownership	− 0.162 (0.183) **	− 0.126 (0.171) **	− 0.119 (0.17) **	− 0.101 (0.163) *	− 0.119 (0.207) *	− 0.089 (0.2)
DT		0.340 (0.047) ***		0.259 (0.046) ***		0.281 (0.055) ***
ERIC			0.365 (0.045) ***	0.287 (0.045) ***		
R^2^	0.215	0.321	0.329	0.385	0.126	0.198
F	16.355	23.738	24.647	27.388	9.089	12.867
Max VIFs	1.087	1.095	1.165	1.273	1.087	1.095

N = 338, *p < 0.05; **p < 0.01; ***p < 0.001.

M1 presents the regression results indicating that several control variables, including to firm size, net profit, government subsidies, R&D intensity and ownership, positively impacts GIP.

M2 expands upon the regression results of M1 by adding independent variables, and the data indicate that DT positively affects GIP (beta = 0.340, *p* < 0.001), in support of H1.

M3 and M4 present regression results for GIP, with M4 controlling for DT. The data indicate that effect of ERIC on GIP (β = 0.287, *p* < 0.001) is significant and positive, in support of H1.

M5 presents the regression results indicating that several control variables have an effect on ERIC.

M6 expands on the results of M5 by adding DT as an independent variable. The findings indicate that the impact of DT on ERIC (β = 0.281, *p* < 0.001) is significant and positive, in support of H2.

Based on model M1–M6, the study hypothesizes that ERIC plays a mediating role in the correlation between DT and GIP. To further test this hypothesis, a bootstrap analysis was performed utilizing the PROCESS software with 5000 bootstrap samples and a confidence interval of 95%. The findings shown in Table 5 indicate that the direct impact of DT on GIP through ERIC is 0.2580, accounting for 76.2% of the total effect, and the confidence interval does not include 0. The mediation effect is 0.0806, accounting for 23.8% of the total effect, and the confidence interval does not include 0. These findings provide further evidence for the significant mediation effect of DT-ERIC-GIP, in support of H4.

**Table 5:** Bootstrap for the mediating effect.

DT-ERIC-GIP Bootstrap					
Item	Coeff	S.E.	5000 times, CI = 95%		Rate (%)
			LLCI	ULCI	
Total	0.3386	0.0466	0.2469	0.4304	100
Direct	0.2580	0.0464	0.1667	0.3492	76.2
Indirect	0.0806	0.0197	0.0486	0.1275	23.8

### Regression analysis of moderating effect

**Figure 2:**
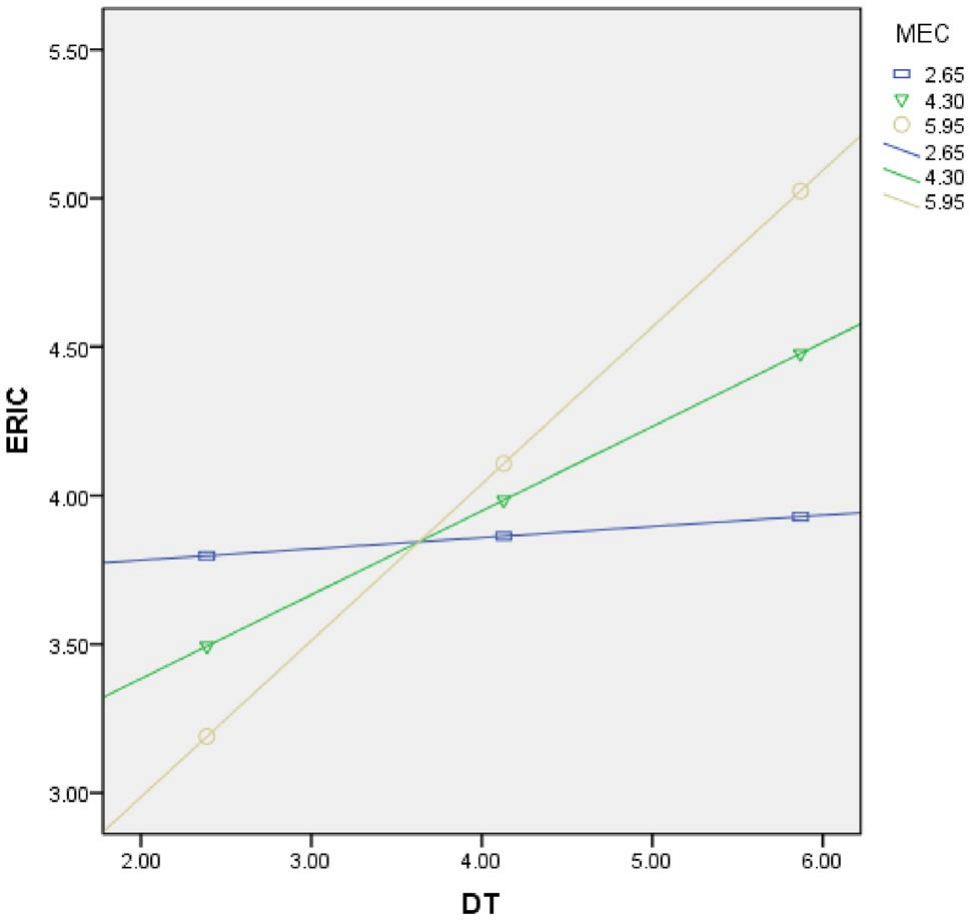
Moderating effect of MEC on DT and ERIC.

**Figure 3:**
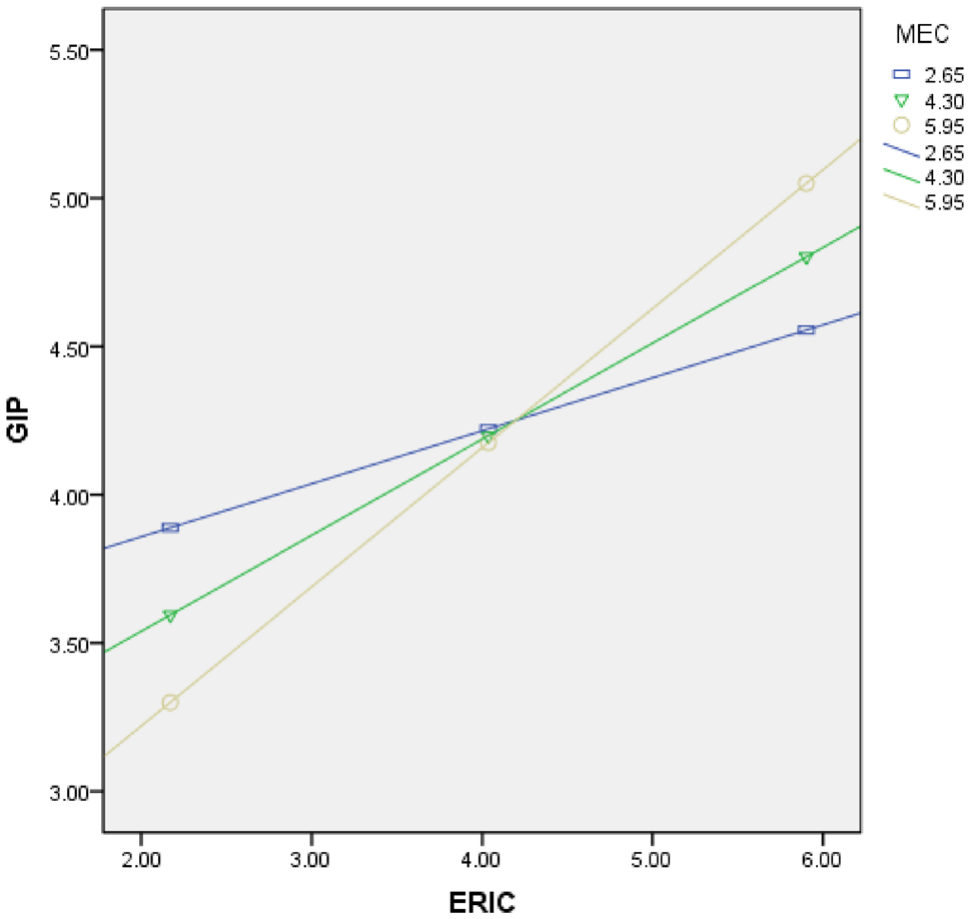
The moderating effect of MEC on ERIC and GIP.

To prevent collinearity caused interaction terms, this research initially carried out decentralized data processing when calculating terms. The findings of the regression analysis of the moderating effect are shown in Table 6. The findings depict that MEC plays a significant positive moderating function in the correlation between DT and ERIC (β = 0.218, *p* < 0.001) as well as ERIC and GIP (β = 0.141, *p* < 0.01). Additionally, to depict the moderating effect, the study employed the PROCESS software to generate a plot, as shown in Figs. 2 and 3. These figures illustrate that when MEC increases by one standard deviation, the slope of DT's effect on ERIC becomes steeper, and the slope of ERIC's effect on GIP also becomes steeper. Conversely, when MEC decreases by one standard deviation, the corresponding slopes both decrease. These findings provide further evidence for the significant moderating effect of MEC, supporting both hypothesis H5 and H6.

**Table 6:** Regression analysis of adjustment effect.

Variables	Dependent variable: ERIC		Dependent variable: GIP	
	M7	M8	M9	M10
Age	− 0.039 (0.065)	− 0.025 (0.063)	0.013 (0.055)	0.005 (0.055)
Size	0.068 (0.07)	0.051 (0.068)	0.179 (0.06) ***	0.166 (0.059) ***
Np	0.123 (0.069) *	0.096 (0.068)	0.113 (0.059) *	0.109 (0.059) *
RD	0.127 (0.066) *	0.125 (0.064) *	0.069 (0.057)	0.062 (0.056)
FS	0.123 (0.065) *	0.110 (0.064) *	0.137 (0.056) **	0.144 (0.055) **
Ownership	− 0.089 (0.199)	− 0.073 (0.194)	− 0.119 (0.17) **	− 0.114 (0.168) *
DT	0.273 (0.055) ***	0.263 (0.053) ***		
ERIC			0.367 (0.045) ***	0.348 (0.045) ***
MEC	0.077 (0.056)	0.065 (0.054)	− 0.015 (0.048)	− 0.013 (0.047)
DT*MEC		0.218 (0.033) ***		
ERIC*MEC				0.141 (0.028) **
R^2^	0.201	0.245	0.328	0.345
F	11.609	13.155	21.523	20.737
Max VIFs	1.095	1.103	1.179	1.198

N = 338, *p < 0.05; **p < 0.01; ***p < 0.001.

### Moderated mediation effects

To further explore the possible mediating effect in the model, this study utilized the PROCESS software, selecting 5000 bootstrap samples and a 95% confidence interval. Running M58 in the PROCESS software yielded the conditional indirect effect value of the overall model in Fig. 1, obtained the mediating effect value of DT-ERIC-GIP, as shown in Table 7.

**Table 7:** Bootstrap for the moderating mediation effect.

Moderator Range	Coeff	S.E	LLCI	ULCI
Lower MEC	0.007	0.015	− 0.020	0.041
Middle MEC	0.075	0.019	0.044	0.118
Higher MEC	0.184	0.045	0.105	0.280
Moderating mediation effect (running M7 in PROCESS)	0.040	0.012	0.021	0.066

The findings depict that the lower the level of MEC, the mediating effect of ERIC is not significant (confidence interval includes 0); However, the mediating role of ERIC becomes significant as MEC increases gradually (confidence interval does not include 0). This suggests that MEC has a positive effect on the mediating function of ERIC. The mediating impact value is 0.040, with an interval of [0.021, 0.066], excluding 0. These findings further indicate that MEC significantly mediates DT-ERIC-GIP. The Moderated mediation effects in the model is exist.

## Conclusion and discussion

### Conclusion

The rapid development of digital transformation has led to its widespread adoption across industries, making it a hot research topic and direction in various fields. In the medical industry, increasing attention is being given to the potential influence of DT on the GIP of medical equipment suppliers. Nonetheless, empirical research is lacking, particularly in the efficacy of environmental resource integration capability and managerial environmental concern. This paper aims to fill this gap by using data of Chinese medical equipment suppliers to verify relevant research hypothesis.

This study finds that digital transformation has a crucial role to play in enhancing green innovation performance through environmental resource integration capability. Additionally, there exists a significant double moderating impact of managerial environmental concern on the relationship of DT-ERIC and ERIC-GIP. These findings not only provide compelling insights into the impact mechanism of digital transformation on the performance of green innovation but also have important implications for relevant academic research and policy-making.

### Theoretical implications

This study’s main contributions are threefold.

Key variable measurement innovation. Firstly, with respect to the resource-based view, it builds on the environmental resource integration capability and green innovation performance measurement of variables, providing a valuable reference for subsequent research. Secondly, it defines environmental resource integration capability as the ability to identify, select, configure and utilize resources related to green production or environmental protection. The study covers the concept and measured results of existing theories and enterprise research viewpoints and passed the reliability and validity test. This study can be used as a reference for measuring these important variables.

This study also demonstrates how the resource-based perspective helps us understand the ways in DT impacts the GIP of medical equipment providers by examining their internal influence mechanisms. Current studies have extensively explored the impact of digital transformation on software technology and software industry^48^, proposed research approaches for the future of digital transformation^37^, and determined the stage of digital transformation^49^, etc. These studies focus on technology, research approaches and other aspects, and ignore the impact of environmental resources integration and managerial environmental concern factors. In addition, there are few discussions about the green innovation performance of medical equipment supply enterprises. In fact, human life and all sectors of society are inseparable from the green innovation and sustainable development of medical equipment supply enterprises. It is necessary for us to explore the impact mechanism. This study is therefore an important supplement to the current green innovation performance- related research.

Finally, based on the RBV, the research reveals the role of managerial environmental concern as a moderator and identifies effective conditions for the relationships between DT-ERIC-GIP. The digital transformation and environmental resource integration capability of medical equipment supply enterprises provide a channel for them to acquire environmental protection knowledge, thus promoting their green innovation performance. However, these kinds of knowledge may not be integrated and systematic (Ramadani et al., 2018), which often leads to problems such as digital transformation and knowledge transfer of environmental resource integration capability is not smooth and ineffective. Given the limitations of current research, the introduction of managerial environmental concern as a moderating variable helps break through barriers of knowledge transfer. The strengthening effect of managerial environmental concern on DT-ERIC-GIP relationship is discussed from the RBV perspective. Furthermore, the study extends the application boundaries of managerial environmental concern and RBV in the field of digital transformation and medical equipment supply.

### Management implications

The study provides two key management insights:

Firstly, the findings underscore the crucial function of digital transformation in enhancing the green innovation performance of medical equipment suppliers. This indicates that medical equipment suppliers should effectively utilize policies and resources related to digital transformation and digital technology to effectively promote environmental resource integration capability and foster managerial environmental concern. This can be achieved through measures such as bulk purchasing of high-value consumables, encouraging innovative medical instrument production modes, promoting research and development of medical equipment, establishing industry service platforms, streamlining emergency examination and approval procedures, and standardizing consumables management policies and protection principles. These measures can effectively enhance green innovation performance and foster sustainable development of medical equipment supply enterprise.

Secondly, the study shows that MEC moderates and mediates the correlation between DT, ERIC and GIP. This suggests that medical equipment supply enterprises’ MEC positively moderating the relationship in DT-ERIC and ERIC-GIP. Therefore, it is important to incorporate managerial environmental concern as a key component in policy, strategy and decision-making processes that moderate DT, ERIC and GIP. In the process of digital transformation, providing extensive training on green, innovative and sustainable development to managers at all levels of medical equipment supply and organizing digital knowledge exchange and experience sharing among enterprises can further promote the willingness of medical equipment suppliers to enhance their green innovation performance.

### Limitation

This study has made significant contributions in various aspects, including variable design, internal mechanism exploration and theoretical breakthrough. There are however certain limitations that need to be acknowledged.

The first limitation is the sample. The data collected for this research is restricted to the management of medical equipment supply enterprises, which only covers activities at the enterprise level. Factors such as relevant regulatory agencies of enterprises, medical workers’ subjective initiative and workers’ cognitive level is not included in the research samples. Future studies can expand the sample size and include data from other relevant institutions to obtain more comprehensive and multi-level research conclusions.

The second limitation is the variable design. The environmental resource integration capability and green innovation performance variables are innovative, but they still have reliability risks. Despite considering the results of relevant research and enterprise investigations in the process of index design, strong subjectivity remains a limitation. Further scientific demonstration is needed to test the practicality of these variables repeatedly and optimize the detection objectives for more robust and accurate measurement results.

Finally, the limitations of theoretical exploration should be acknowledged. The practice of the medical equipment supply enterprises is complex and the influence of the specific situation may be more diverse than just digital transformation on green innovative performance. This study is limited to the resource-based view research framework. Further studies can explore other perspectives to obtain a broader research conclusion.

## Data Availability

The datasets used of this study is available from the corresponding author on reasonable request.

## References

[1] Nagel L (2020). The influence of the COVID-19 pandemic on the digital transformation of work. Int. J. Sociol. Soc. Policy.

[2] Warner KS, Wäger M (2019). Building dynamic capabilities for digital transformation: An ongoing process of strategic renewal. Long Range Plan..

[3] Carcary, M., Doherty, E. & Conway, G. in *The European Conference on Information Systems Management.* 20 (Academic Conferences International Limited).

[4] Mo X, Boadu F, Liu Y, Chen Z, Ofori AS (2022). Corporate social responsibility activities and green innovation performance in organizations: Do managerial environmental concerns and green absorptive capacity matter?. Front. Psychol..

[5] Rassool MR, Dissanayake DR (2019). Digital transformation for small & medium enterprises (Smes): With special focus on Sri Lankan context as an emerging economy. Int. J. Bus. Manage. Rev..

[6] Nambisan S, Wright M, Feldman M (2019). The digital transformation of innovation and entrepreneurship: Progress, challenges and key themes. Res. Policy.

[7] Hinings B, Gegenhuber T, Greenwood R (2018). Digital innovation and transformation: An institutional perspective. Inf. Organ..

[8] Chan JO-P (2020). Digital transformation in the era of big data and cloud computing. Int. J. Intell. Inf. Syst..

[9] Yang D, Jiang W, Zhao W (2019). Proactive environmental strategy, innovation capability, and stakeholder integration capability: A mediation analysis. Bus. Strateg. Environ..

[10] García-Sánchez E, García-Morales VJ, Martín-Rojas R (2018). Analysis of the influence of the environment, stakeholder integration capability, absorptive capacity, and technological skills on organizational performance through corporate entrepreneurship. Int. Entrep. Manage. J..

[11] Shen J, Sha Z, Wu YJ (2020). Enterprise adaptive marketing capabilities and sustainable innovation performance: An opportunity–resource integration perspective. Sustainability.

[12] Sun Y, Sun H (2021). Green innovation strategy and ambidextrous green innovation: The mediating effects of green supply chain integration. Sustainability.

[13] Song W, Yu H, Xu H (2020). Effects of green human resource management and managerial environmental concern on green innovation. Eur. J. Innov. Manage..

[14] Saudi MHM, Obsatar Sinaga G, Zainudin Z (2019). The effect of green innovation in influencing sustainable performance: Moderating role of managerial environmental concern. Int. J. Supply Chain Manage..

[15] Yusoff YM, Nejati M, Kee DMH, Amran A (2020). Linking green human resource management practices to environmental performance in hotel industry. Global Bus. Rev..

[16] Seman NAA (2019). The mediating effect of green innovation on the relationship between green supply chain management and environmental performance. J. Clean. Prod..

[17] Hermes S, Riasanow T, Clemons EK, Böhm M, Krcmar H (2020). The digital transformation of the healthcare industry: Exploring the rise of emerging platform ecosystems and their influence on the role of patients. Bus. Res..

[18] Ângelo, A., Barata, J., Cunha, P. R. D. & Almeida, V. *European, Mediterranean, and Middle Eastern Conference on Information Systems* 307–323. (Springer).

[19] Jahankhani H, Kendzierskyj S (2019). Blockchain and Clinical Trial.

[20] Smith, S., Lane, M., Toleman, M. & Shrestha, A. Digital Transformation of the Healthcare Supply Chain: A Clinical Safety Evaluation Model. (2022).

[21] Yahya S, Khan A, Farooq M, Irfan M (2022). Integrating green business strategies and green competencies to enhance green innovation: Evidence from manufacturing firms of Pakistan. Environ. Sci. Pollut. Res..

[22] Du L, Zhang Z, Feng T (2018). Linking green customer and supplier integration with green innovation performance: The role of internal integration. Bus. Strategy Environ..

[23] Albort-Morant G, Leal-Rodríguez AL, De Marchi V (2018). Absorptive capacity and relationship learning mechanisms as complementary drivers of green innovation performance. J. Knowl. Manage..

[24] Yang Z, Lin Y (2020). The effects of supply chain collaboration on green innovation performance: An interpretive structural modeling analysis. Sustain. Prod. Consum..

[25] Du Y, Li Z, Du J, Li N, Yan B (2019). Public environmental appeal and innovation of heavy-polluting enterprises. J. Clean. Prod..

[26] Bhalaji, R. *et al.**Supply Chain Forum: An International Journal.* 1–25 (Taylor & Francis).

[27] Cheng S-L (2019). Comparison between COPD Assessment Test (CAT) and modified Medical Research Council (mMRC) dyspnea scores for evaluation of clinical symptoms, comorbidities and medical resources utilization in COPD patients. J. Formos. Med. Assoc..

[28] Waqas M (2022). Triggering sustainable firm performance, supply chain competitive advantage, and green innovation through lean, green, and agile supply chain practices. Environ. Sci. Pollut. Res..

[29] Mikalef P, Boura M, Lekakos G, Krogstie J (2019). Big data analytics capabilities and innovation: The mediating role of dynamic capabilities and moderating effect of the environment. Br. J. Manage..

[30] Dutta G, Kumar R, Sindhwani R, Singh RK (2020). Digital transformation priorities of India’s discrete manufacturing SMEs—A conceptual study in perspective of Industry 4.0. Compet. Rev. Int. Bus. J..

[31] Fenech, R., Baguant, P. & Ivanov, D. The changing role of human resource management in an era of digital transformation. *J. Manage. Inf. Dec. Sci.* **22** (2019).

[32] Jones P, Wynn M (2021). The leading digital technology companies and their approach to sustainable development. Sustainability.

[33] Khumpang, P. & Arunyanart, S. *IOP Conference Series: Materials Science and Engineering.* 012001 (IOP Publishing).

[34] Kuo F-I, Fang W-T, LePage BA (2022). Proactive environmental strategies in the hotel industry: Eco-innovation, green competitive advantage, and green core competence. J. Sustain. Tourism.

[35] Khurana S, Haleem A, Luthra S, Mannan B (2021). Evaluating critical factors to implement sustainable oriented innovation practices: An analysis of micro, small, and medium manufacturing enterprises. J. Clean. Prod..

[36] Miao C, Fang D, Sun L, Luo Q (2017). Natural resources utilization efficiency under the influence of green technological innovation. Resour. Conserv. Recycl..

[37] Reis, J., Amorim, M., Melão, N. & Matos, P. *World Conference on Information Systems and Technologies.* 411–421 (Springer).

[38] Sun, S. & Guo, L. Digital Transformation, Green Innovation and The Solow Productivity Paradox. (2022). 10.1371/journal.pone.0270928 PMC9269481 35802578

[39] Wu L, Sun L, Chang Q, Zhang D, Qi P (2022). How do digitalization capabilities enable open innovation in manufacturing enterprises? A multiple case study based on resource integration perspective. Technol. Forecast. Soc. Change.

[40] Bocconcelli R (2020). Resource interaction and resource integration: Similarities, differences, reflections. Ind. Market. Manage..

[41] Mengcheng L, Tuure T (2022). Information technology–supported value co-creation and co-destruction via social interaction and resource integration in service systems. J. Strategic Inf. Syst..

[42] Qiu L, Jie X, Wang Y, Zhao M (2020). Green product innovation, green dynamic capability, and competitive advantage: Evidence from Chinese manufacturing enterprises. Corporate Soc. Responsib. Environ. Manage..

[43] Dangelico RM, Pujari D, Pontrandolfo P (2017). Green product innovation in manufacturing firms: A sustainability-oriented dynamic capability perspective. Bus. Strategy Environ..

[44] Yao, Q., Tang, H., Boadu, F. & Xie, Y. Digital transformation and firm sustainable growth: The moderating effects of cross-border search capability and managerial digital concern. *J. Knowl. Econ.*, 1–25 (2022).

[45] Vial G (2019). Understanding digital transformation: A review and a research agenda. J. Strateg. Inf. Syst..

[46] Tang H, Yao Q, Boadu F, Xie Y (2022). Distributed innovation, digital entrepreneurial opportunity, IT-enabled capabilities, and enterprises' digital innovation performance: A moderated mediating model. Eur. J. Innov. Manage..

[47] Xie Y, Boadu F, Tang H (2022). Does internationalization encourage state-owned enterprises to utilize subsidies to innovate? Evidence from high-tech and automobile manufacturing industries of Chinese listed companies. Chin. Manage. Stud..

[48] Ebert C, Duarte CHC (2018). Digital transformation. IEEE Softw..

[49] Verhoef PC (2021). Digital transformation: A multidisciplinary reflection and research agenda. J. Bus. Res..

